# Supplementary material for: Prefrontal reinstatement of contextual task demand is predicted by separable hippocampal patterns
Source: Nat Commun. 2020 Apr 28;11:2053. doi: 10.1038/s41467-020-15928-z (PMC7188806; doi:10.1038/s41467-020-15928-z)
Supplement: Supplementary file 1 — Supplementary Information [file 41467_2020_15928_MOESM1_ESM.pdf]

Supplementary Information

Prefrontal reinstatement of contextual task demand is mediated by separable hippocampal patterns

Jiang et al

Supplementary Figures

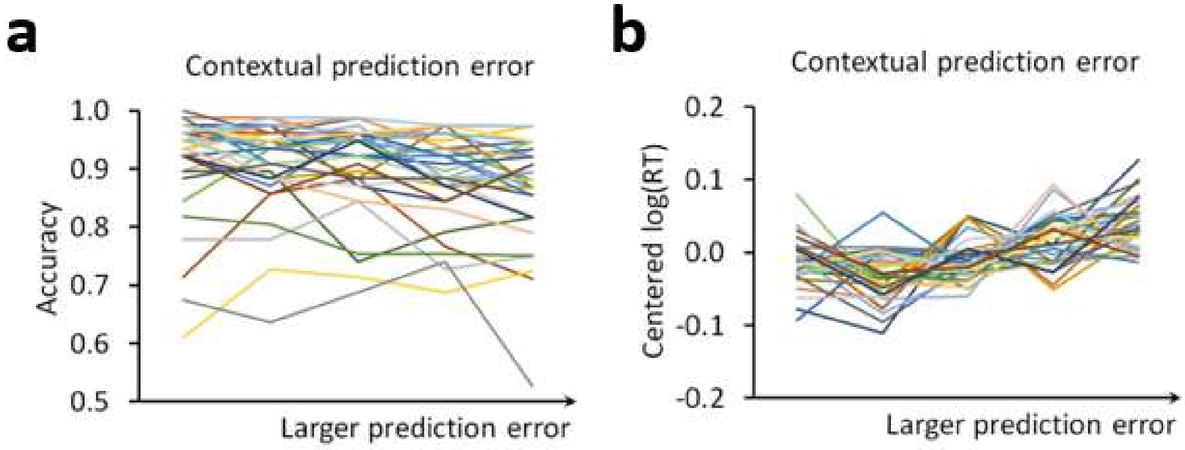

Supplementary Figure 1. Individual quintile data corresponding to Fig. 3b (a) and 3c (b). Source data are provided as a Source Data file.

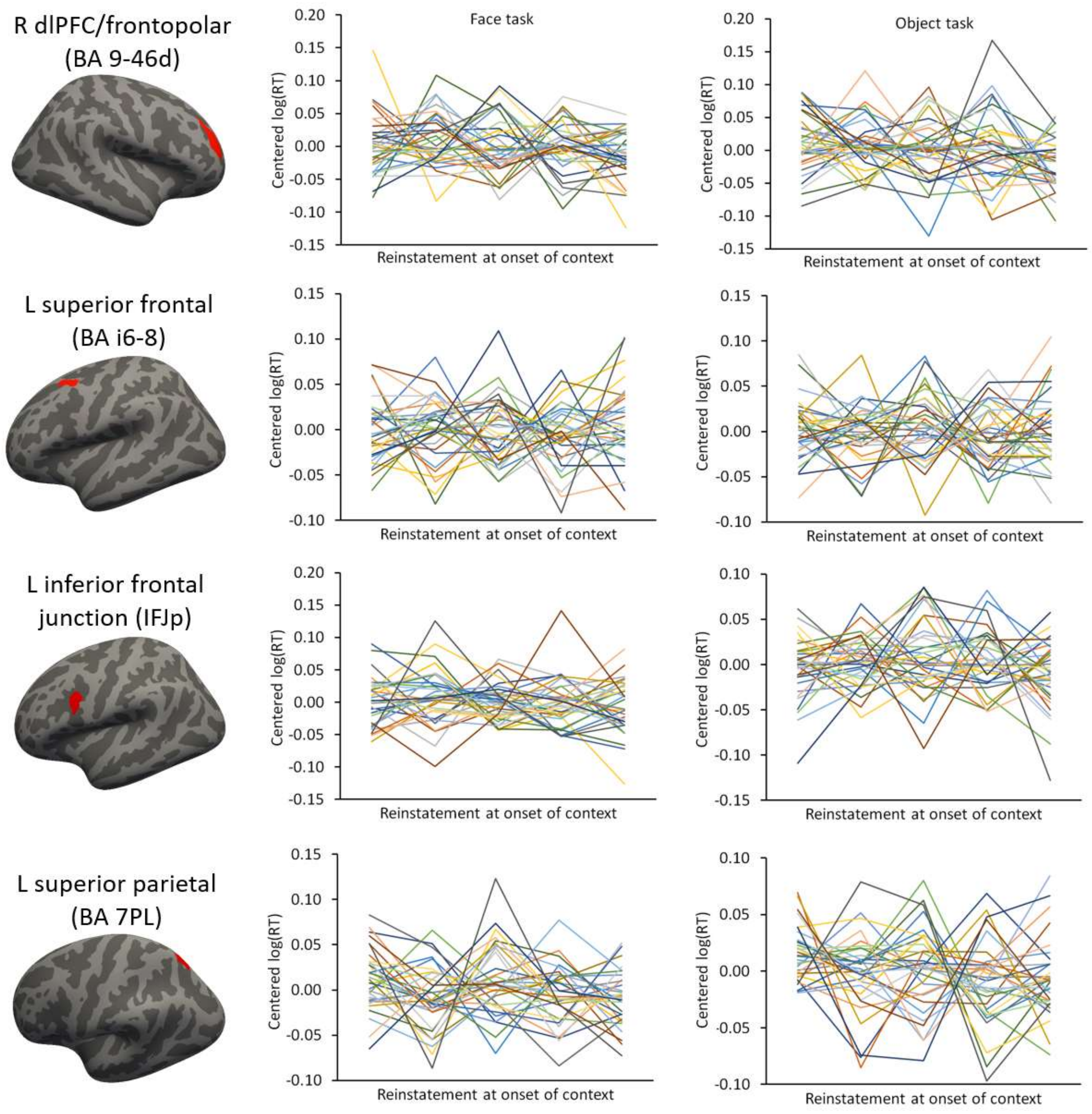

Supplementary Figure 2. Individual quintile data corresponding to Fig. 4c-f. Source data are provided as a Source Data file.

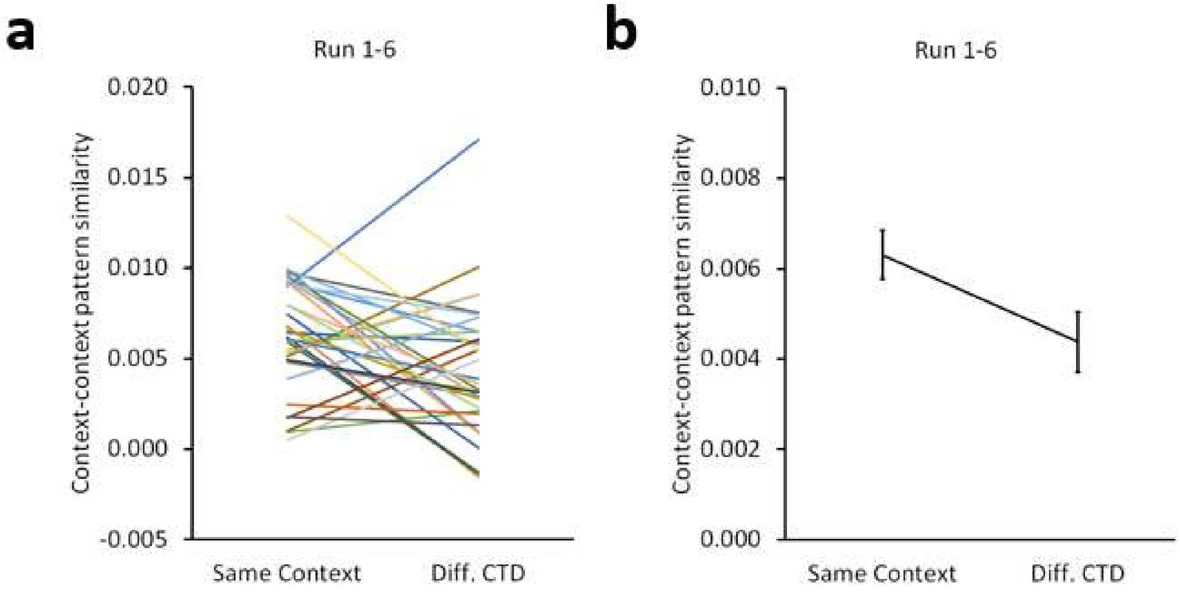

Supplementary Figure 3. Individual (a) and mean ± SEM of (b) context-context pattern similarity for Same Context and Different CTD conditions. Each line represents one participant. Source data are provided as a Source Data file.

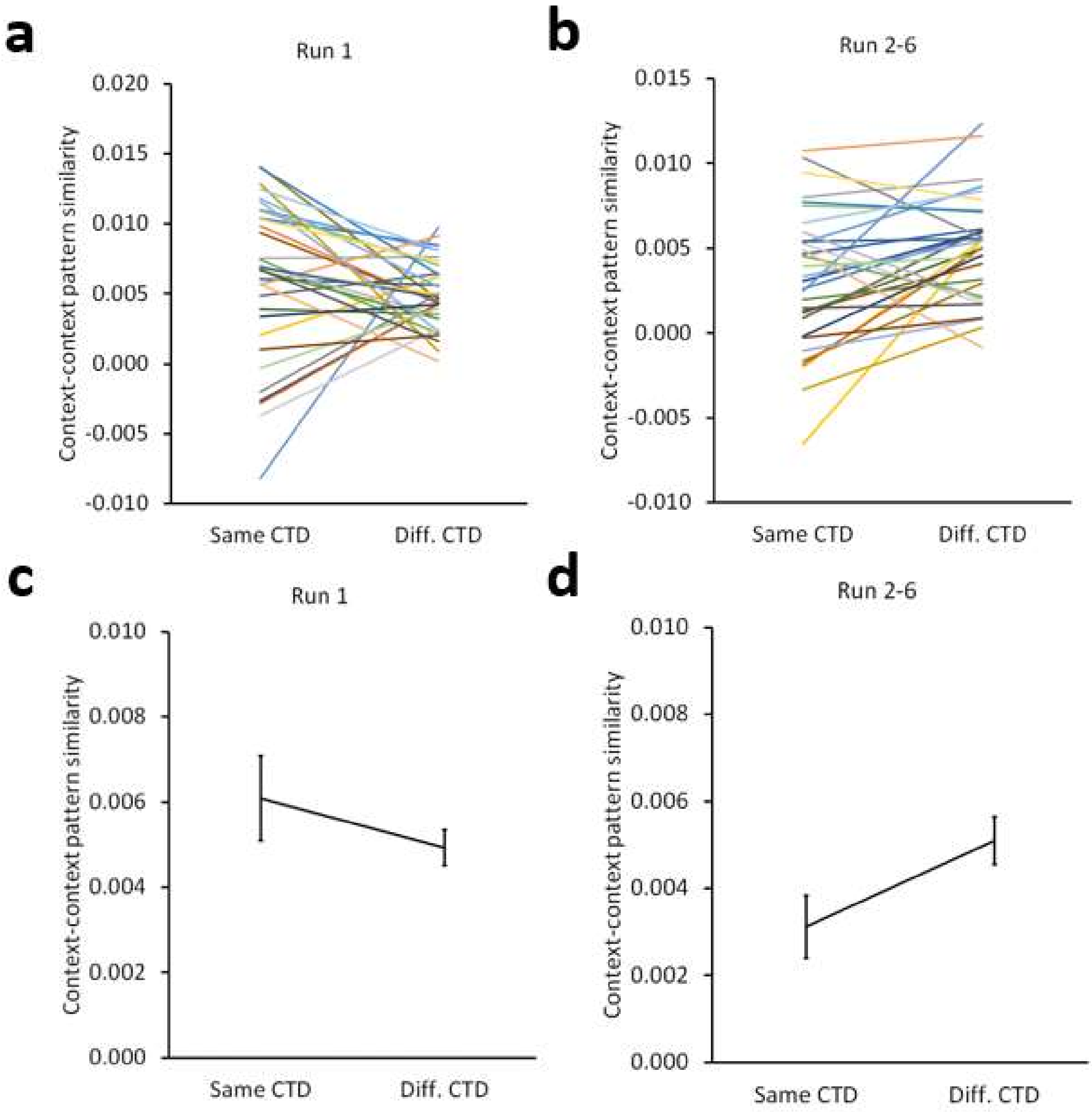

Supplementary Figure 4. Individual (a-b) and mean ± SEM of (c-d) context-context pattern similarity, plotted as a function of experimental condition (Same Context and Different CTD) and time (run 1 and runs 2-6). Each line represents one participant. Source data are provided as a Source Data file.

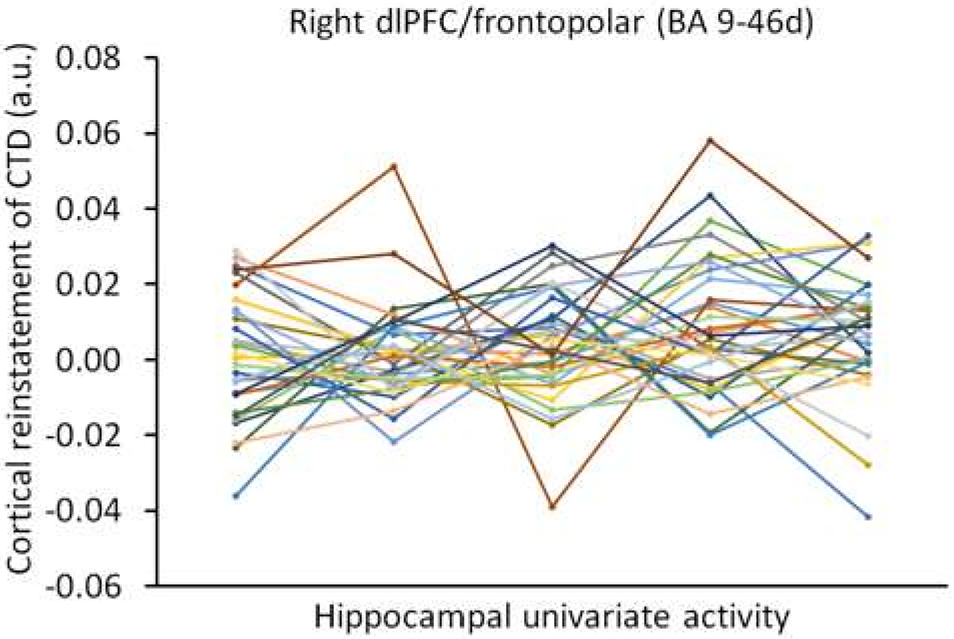

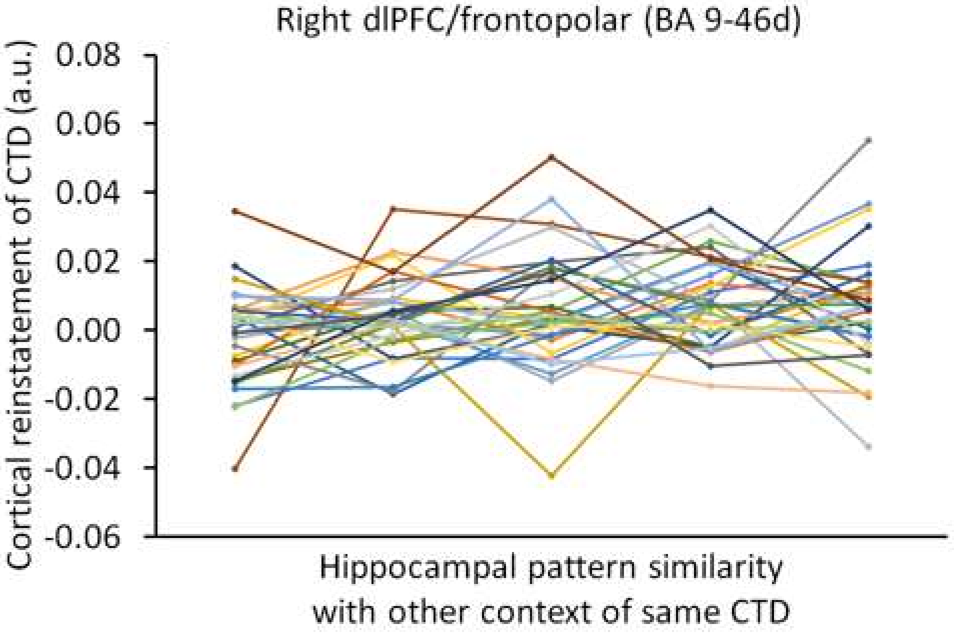

Supplementary Figure 5. Individual quintile data corresponding to Fig. 5d. Source data are provided as a Source Data file.

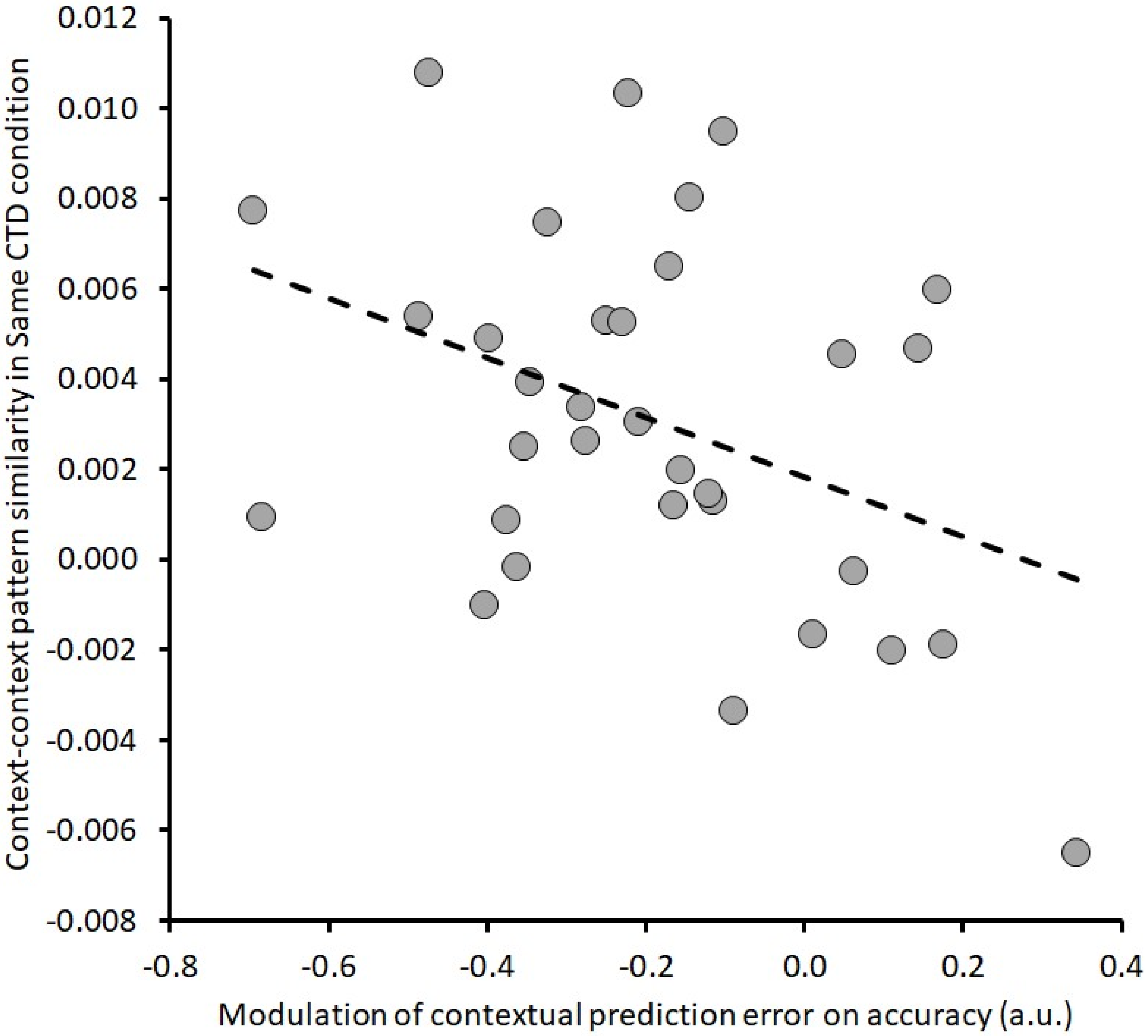

Supplementary Figure 6. Individual modulation of contextual prediction error on accuracy (lower value indicates stronger modulation), plotted as a function of the Same CTD context-context pattern similarity in the hippocampus (lower value indicates stronger separation). Dashed line represents linear trend line. Source data are provided as a Source Data file.

Supplementary Notes

Supplementary Note 1: Unique contributions of temporal and contextual prediction error

Given that temporal prediction error and contextual prediction error are correlated ($r = 0.44 \pm 0.04$, range: 0.10-0.87), we replicated the previous behavioral analyses using residuals of contextual prediction error after regression on the temporal prediction error, and obtained qualitatively similar results for both accuracy (regression coefficient: -0.19 ± 0.04; $t_{32} = -4.52$; $P < 0.001$, one-sample t-test; $d = 0.79$) and RT (regression coefficient: 0.01 ± 0.004; $t_{32} = 2.64$; $P = 0.01$, one-sample t-test; $d = 0.46$). In addition, after regressing shared variance with contextual prediction error, temporal prediction error still exhibited significant modulation on trial-level RT (regression coefficient: 0.03 ± 0.004; $t_{32} = 9.29$; $P < 0.001$, one-sample t-test; $d = 1.62$).

Supplementary Note 2: CTD reinstatement in visual cortex

To examine the reinstatement of CTD in the visual cortex, we tested the interaction between context and congruency (Fig. 4b) in 34 visual ROIs (defined by the multi-modal cortical parcellation from the Human Connectome Project; major assignment IDs: 1, 2, 3, and 4; ROI size = 269 ± 52 voxels; range = 49–1183)[10]. After correction for multiple comparisons, only the left V8 ROI exhibited a significant context × congruency interaction effect ($t_{32} = 3.69$, $P < 0.001$). However, the reinstatement of CTD at the beginning of a block in left V8 did not modulate behavior later in the block ($t_{32} = 0.80$, $P = 0.43$). Given that the right dlPFC/frontopolar ROI exhibited both a context × congruency interaction and modulation of CTD reinstatement on behavior, the lack of visual areas showing both effects suggests that the prefrontal reinstatement of CTD is not solely explained by perceptual facilitation.

Supplementary Note 3: CTD reinstatement in medial prefrontal cortex

Given the importance of the medial prefrontal cortex in context-dependent memory retrieval and generalization, we compared the context-context pattern similarity in the Same CTD condition with the Different CTD condition in each of the 32 ROIs in the 'ACC and medial prefrontal cortex' category (ROI size = 166 ± 30 voxels; range = 61–446) of the multi-modal cortical parcellation from the Human Connectome Project. Only the left pOFC area exhibited a significant reduction of context-context pattern similarity in the Same CTD than the Different CTD condition, and this was true only when using an uncorrected alpha-level (difference: 0.0035 ± 0.0017, $t_{32} = -2.11$; uncorrected $P = 0.043$, paired t-test; $d = 0.37$). We then tested the prediction of the Same CTD condition context-context pattern similarity on the CTD reinstatement in the right dlPFC (see Fig. 5c) for each of the ROIs, but did not find any ROI showing the positive prediction that

was observed for the hippocampus (all $P > 0.051$). These results suggest a limited involvement of the medial prefrontal cortex in retrieval of the CTD.

Supplementary Note 4: CTD reinstatement in motor and premotor cortex
To examine whether and how retrieval of the CTD predicts motor planning and response preparation, we tested the univariate activity at the onset of the context in each of the 12 premotor, somatosensory, and motor cortex ROIs (major assignment IDs 6 and 8 in the multi-modal cortical parcellation from the Human Connectome Project; collapsed across left and right hemispheres; ROI size = 371 ± 32 voxels; range = 71–1106). The univariate activity level was defined as the ROI-mean of the multi-voxel normalized betas[9], in order to be consistent with the pattern similarity analyses. Given that participants used different hands for the two tasks in our experimental design, retrieval of the CTD predicts increased univariate activity in motor/premotor areas contralateral to the hand used for the task predicted by the CTD, as compared to the ipsilateral hand. Although we observed such a pattern in the L_1 area when using an uncorrected alpha-level (contralateral - ipsilateral: 0.065 ± 0.027, $t_{32} = 2.43$; uncorrected $P = 0.021$, paired t-test; $d = 0.42$), no ROI survived the FDR correction.

Furthermore, to test whether motor and premotor reinstatement of CTD in the activity patterns at the context onset affects behavior later in the block, we tested the CTD reinstatement modulation on RT (using an identical procedure to that used with the frontoparietal ROIs) in each of the aforementioned 24 ROIs. None of the ROIs showed significant behavioral modulation (all $Ps > 0.09$). Taken together, we did not observe strong evidence for response bias at the context onset.

Supplementary Note 5: Analysis of temporal change in context-context pattern similarity
We examined whether there was a systematic temporal change in context-context pattern similarity by calculating the correlation between block-level pattern similarity measures (each of the Same context, Same CTD and Different conditions) and time (each of block ID and the logarithm of block ID) for each participant. One sample t-tests against 0 at the group level did not reveal any significant temporal change that was consistent across participants (all $Ps > 0.56$).

Supplementary Note 6: Emergence of hippocampal pattern differentiation
A key assumption of hippocampal pattern differentiation is that the differentiation occurs, potentially relatively rapidly, through learning. To test this, we calculated context-context pattern similarity between run 1 and runs 2-6 and compared it to the context-context pattern similarity calculated within runs 2-6. Consistent with the pattern differentiation analysis in Fig. 5b, the comparison was performed between the Same

CTD and the Different CTD conditions. To test whether the difference in context-context pattern similarity evolves through time, we conducted a repeated-measures 2 (condition: Same CTD/Different CTD) × 2 (time: run 1/run 2-6) ANOVA, which revealed a significant interaction between condition and time ($F_{1,32}$=8.30, P = 0.007). Post-hoc analysis showed that in run 1, context-context pattern similarity was not significantly different between the Same CTD (0.0061 ± 0.0010) and the Different CTD condition (0.0049 ± 0.0004, t_{32} = 1.10; P = 0.28; paired t-test; d = 0.19; Supplementary Fig. 4), whereas in runs 2-6, pattern differentiation was observed (Same CTD: 0.0031 ± 0.0008; Different CTD: 0.0051 ± 0.0005; t_{32} = -2.91; P < 0.01, paired t-test; d = 0.51; Fig. 5b and Supplementary Fig. 4). Importantly, this temporal emergence of the pattern differentiation effect is driven by the decrease of context-context pattern similarity in the Same CTD condition (t_{32} = 2.54; P = 0.02), whereas the similarity was constant across the six scan runs in the Different CTD condition (t_{32} = -0.80; P = 0.43). Taken together, these results support the conclusion that hippocampal pattern differentiation increased through time.

Supplementary Note 7: Directionality of hippocampal prediction of CTD reinstatement in dlPFC

One alternative interpretation of the results is that hippocampal pattern separation is mediated by CTD reinstatement in the dlPFC. If this were true, one might predict that the dlPFC reinstatement would be modulated by other learning systems. We tested this prediction by repeating the analyses in the striatum (defined using the labels caudate, putamen and accumbens areas from FreeSurfer). CTD reinstatement in dlPFC was not significantly modulated by striatal univariate activity nor by striatal context-context pattern similarity in any of the Same Context, Same CTD, or Different CTD conditions (all Ps > 0.33). This result lends indirect support to the argument that CTD reinstatement in the dlPFC is modulated by hippocampal activity and pattern differentiation.

An additional possibility is that both the dlPFC reinstatement of CTD and the hippocampal context-context pattern similarity in the Same CTD condition are mediated by a common modulator. To test this hypothesis, we searched for potential common modulators in the 359 cortical ROIs (excluding right BA 9-46d) defined by the multi-modal cortical parcellation from the Human Connectome Project. Specifically, for each ROI, its univariate activity (which may vary with general load, surprise, or novelty) and context-trial pattern similarity in the Same Context (reflecting stimulus-level content representation) and the Same CTD (reflecting task demand-level representation) were used to explain variance of both the dlPFC reinstatement of CTD and the hippocampal context-context pattern similarity in the Same CTD condition at the block level. Individual modulation strength was then tested against 0 at the group level using one-sample t-test. Multiple comparisons were leniently corrected using FDR within each

ROI, in order to reduce type II error. A common modulator would show significant modulation in at least one of its three measures above on both dlPFC reinstatement of CTD and hippocampal pattern differentiation. However, in contrast to a common modulator hypothesis, no ROI met the criteria. However, as this is a null result, interpretative caution is warranted. Future studies are encouraged to further examine the directionality of the relationship between dlPFC reinstatement of CTD and hippocampal activity patterns at retrieval.

Supplementary Note 8: Joint behavioral model

To quantitatively assess the joint contribution of temporal and contextual predictions of task demand to behavior, we designed an additional model, which combines both predictions and takes the form:

$$P_o(t) = w*P_o^c(t) + (1-w)*P_o^t(t)$$

Where P_o is the joint prediction of task demand, which is a weighted sum of P_o^c and P_o^t (denoting the contextual and temporal predictions of task demand [see Methods: Behavioral Analysis], respectively). The model includes three free parameters (the weight w and the respective learning rates for P_o^c and P_o^t), which were determined using a grid search (w range: 0-1, step size = 0.01; learning rate range: 0.01-0.99, step size = 0.01) that maximizes the variance explained in the trial-wise RT data (see Methods: Behavioral Analysis). At the group level, the weight was significantly lower than 0.5 ($w = 0.30 \pm 0.05$, $t_{32} = 4.20$; $P < 0.001$, one-sample t-test; $d = 0.73$), indicating that subjects rely more on temporal than contextual predictions. Furthermore, the joint prediction error significantly modulated accuracy (regression coefficient: -0.24 ± 0.04; $t_{32} = -5.72$; $P < 0.001$, one-sample t-test; $d = 1.00$) and RT (regression coefficient: 0.04 ± 0.003; $t_{32} = 13.53$; $P < 0.001$, one-sample t-test; $d = 2.36$)."
