# Supplementary material for: Prefrontal reinstatement of contextual task demand is predicted by separable hippocampal patterns
Source: Nat Commun. 2020 Apr 28;11:2053. doi: 10.1038/s41467-020-15928-z (PMC7188806; doi:10.1038/s41467-020-15928-z)
Supplement: Supplementary file 3 — Reporting Summary [file 41467_2020_15928_MOESM3_ESM.pdf]

nature research

Corresponding author(s): Jiefeng Jiang

Last updated by author(s): Mar 16, 2020

Reporting Summary

Nature Research wishes to improve the reproducibility of the work that we publish. This form provides structure for consistency and transparency in reporting. For further information on Nature Research policies, see Authors & Referees and the Editorial Policy Checklist.

Statistics

For all statistical analyses, confirm that the following items are present in the figure legend, table legend, main text, or Methods section.

n/a	Confirmed	
☐	☒	The exact sample size (n) for each experimental group/condition, given as a discrete number and unit of measurement
☐	☒	A statement on whether measurements were taken from distinct samples or whether the same sample was measured repeatedly
☐	☒	The statistical test(s) used AND whether they are one- or two-sided<br>*Only common tests should be described solely by name; describe more complex techniques in the Methods section.*
☐	☒	A description of all covariates tested
☐	☒	A description of any assumptions or corrections, such as tests of normality and adjustment for multiple comparisons
☐	☒	A full description of the statistical parameters including central tendency (e.g. means) or other basic estimates (e.g. regression coefficient) AND variation (e.g. standard deviation) or associated estimates of uncertainty (e.g. confidence intervals)
☐	☒	For null hypothesis testing, the test statistic (e.g. F, t, r) with confidence intervals, effect sizes, degrees of freedom and P value noted<br>*Give P values as exact values whenever suitable.*
☒	☐	For Bayesian analysis, information on the choice of priors and Markov chain Monte Carlo settings
☒	☐	For hierarchical and complex designs, identification of the appropriate level for tests and full reporting of outcomes
☐	☒	Estimates of effect sizes (e.g. Cohen's d, Pearson's r), indicating how they were calculated

Our web collection on statistics for biologists contains articles on many of the points above.

Software and code

Policy information about availability of computer code

Data collection	Python 2.7, Panda EPL
Data analysis	Matlab 2017a, SPM 12, fMRIprep 1.1.4, custom Matlab scripts (https://github.com/JiefengJiang/CTD)

For manuscripts utilizing custom algorithms or software that are central to the research but not yet described in published literature, software must be made available to editors/reviewers. We strongly encourage code deposition in a community repository (e.g. GitHub). See the Nature Research guidelines for submitting code & software for further information.

Data

Policy information about availability of data

All manuscripts must include a data availability statement. This statement should provide the following information, where applicable:
- Accession codes, unique identifiers, or web links for publicly available datasets
- A list of figures that have associated raw data
- A description of any restrictions on data availability

Raw MRI data and behavioral data can be downloaded at [https://openneuro.org/datasets/ds002169]

Field-specific reporting

Please select the one below that is the best fit for your research. If you are not sure, read the appropriate sections before making your selection.

☐ Life sciences ☒ Behavioural & social sciences ☐ Ecological, evolutionary & environmental sciences

For a reference copy of the document with all sections, see nature.com/documents/nr-reporting-summary-flat.pdf

Behavioural & social sciences study design

All studies must disclose on these points even when the disclosure is negative.

Study description	Quantitative experimental
Research sample	The final sample consisted of 33 participants (18–32 yrs old; 19 females, 13 males and 1 NA) with normal or corrected-to-normal vision and no self-reported history of psychiatric or neurological disorders. Subjects came from Stanford University and nearby areas.
Sampling strategy	No sample-size calculation was performed. The sample size was determined based on previous fMRI studies of context-control demand association (King et al., 2012, N=30), item-control demand association (Chiu et al., in revision, N=28) and memory reinstatement (Gordon et al., 2014, N=33).
Data collection	Behavioral data was collected using MR-compatible button boxes. MRI data were acquired on a 3T GE Discovery MR750 MRI scanner (GE Healthcare) using a 32-channel radiofrequency receive-only head coil (Nova Medical).
Timing	05/2018-08/2018
Data exclusions	Another two subjects were excluded due to low behavioral performance (accuracy was lower than the group median minus 3 standard deviation). An additional subject was excluded due to excessive head motion.
Non-participation	Two subjects dropped out before the experiment ended (one felt motion sick and one felt anxious).
Randomization	This study uses a within subject design. Individual difference is treated as random effect.

Reporting for specific materials, systems and methods

We require information from authors about some types of materials, experimental systems and methods used in many studies. Here, indicate whether each material, system or method listed is relevant to your study. If you are not sure if a list item applies to your research, read the appropriate section before selecting a response.

Materials & experimental systems

n/a	Involved in the study
☒	☐ Antibodies
☒	☐ Eukaryotic cell lines
☒	☐ Palaeontology
☒	☐ Animals and other organisms
☐	☒ Human research participants
☒	☐ Clinical data

Methods

n/a	Involved in the study
☒	☐ ChIP-seq
☒	☐ Flow cytometry
☐	☒ MRI-based neuroimaging

Human research participants

Policy information about studies involving human research participants

Population characteristics	The final sample consisted of 33 participants (18-32 yrs old; 19 females, 13 males and 1NA) with normal or corrected-to-normal vision and no self-reported history of psychiatric or neurological disorders.
Recruitment	Subjects were recruited through Stanford's SONA system. There were no apparent biases in recruiting that would impact the findings
Ethics oversight	Thirty-eight subjects gave informed written consent, in accordance with procedures approved by the Stanford University Institutional Review Board.

Note that full information on the approval of the study protocol must also be provided in the manuscript.

Magnetic resonance imaging

Experimental design

Design type	task-state fMRI, event-related and block-level design
Design specifications	Participants performed a perceptual decision-making paradigm (Fig. 2A) embedded in a spatial navigation task in a 3D environment made using Python and Panda EPL. The environment consisted of a circular track and four visually distinct buildings (Fig. 2C) located on the exterior side of the track. The experiment was divided into 6 runs of 8 blocks each, with 2 blocks/building/run. Each block started with a building cue for 6s, indicating to which building the participant was

to navigate. The participant then moved on the circular track to the cued building and indicated their arrival by a button press. If the response was not made at the entrance to the cued building, an error message was presented for 1s. On error trials, the participant resumed navigating until the correct building was reached.

Immediately upon arriving at the cued building, the interior of a room in the building was presented for 7.75s (Fig. 2A). The colors and textures of the backwall and the floor were unique for each room, thus creating four distinct spatial contexts — defined by the perceptual features of the room along with which building the room was located. All four contexts included a display on the backwall. For each block within a room, participants performed eight perceptual decision-making trials presented on the display on the backwall. Each trial began with the presentation of a task cue (either the letter G or O) for 250ms, followed by the presentation of overlapping, translucent images of a face (either male or female) and an object (either clothes or tool) for 1500ms. The particular combinations of face and object images were randomly generated without repetition for each participant. Depending on the cue, participants were required to categorize either the gender of the face (cued by letter G) or the type of the object (cued by letter O), by pressing one of two response buttons (response mappings are shown in Fig. 2A). Trials were separated by a 4.25s inter-trial interval. Critically, to induce different learned associations between each context and expected task demands (face vs. object) — i.e., the CTD — blocks consisted of 75% face/25% object trials in two contexts and 75% object/25% face trials in the other two contexts. The pairings of contexts and CTD were randomized across participants.

Behavioral performance measures: Accuracy and response time on each trial

Acquisition

Imaging type(s): functional, structural

Field strength: 3.0T

Sequence & imaging parameters: Functional data were acquired using a 3-band echo planar imaging (EPI) sequence (acceleration factor = 2) consisting of 63 oblique axial slices parallel to the long axis of the hippocampus (TR = 2 s, TE = 30 ms, flip angle = 74°, FOV = 215 mm × 215 mm, voxel size = 1.8 × 1.8 × 1.8 mm3). To correct for distortions of the B0 field that may occur with EPI, we collected two B0 field maps before every functional run, one in each phase encoding direction, with the same slice prescription as the functional runs. Structural images were acquired using a T1-weighted (T1w) spoiled gradient recalled echo structural sequence (186 sagittal slices, slice thickness = 0.9 mm, TR = 7.26 ms, FoV = 230 mm × 230 mm, in-plane resolution = 0.9 mm × 0.9 mm).

Area of acquisition: whole-brain

Diffusion MRI: ☐ Used ☒ Not used

Preprocessing

Preprocessing software: fMRIprep 1.1.4

Normalization: Spatial normalization to the ICBM 152 Nonlinear Asymmetrical template version 2009c (RRID:SCR_008796) was performed through nonlinear registration with antsRegistration (ANTs 2.2.0, RRID:SCR_004757), using brain-extracted versions of both the T1w volume and template.

Normalization template: ICBM 152 Nonlinear Asymmetrical template version 2009c (RRID:SCR_008796)

Noise and artifact removal: A deformation field, to correct for susceptibility distortions, was estimated based on two EPI references with opposing phase-encoding directions, using 3dQwarp (AFNI). Based on the estimated susceptibility distortion, an unwarped BOLD reference was calculated enabling a more accurate co-registration with the anatomical reference. Head-motion parameters with respect to the BOLD reference (transformation matrices, and six corresponding rotation and translation parameters) were estimated before spatiotemporal filtering using MCFLIRT (FSL 5.0.9).

Volume censoring: Volumes with DVARS greater than 5 and frame-wise translation greater than 0.9mm were excluded from further analysis.

Statistical modeling & inference

Model type and settings: RSA

Effect(s) tested: Reinstatement of contextual task demand (CTD), modulation of CTD reinstatement on response time, and the modulation of hippocampal pattern separation on CTD reinstatement.

Specify type of analysis: ☐ Whole brain ☒ ROI-based ☐ Both

Anatomical location(s): Hippocampus, cortical ROIs defined by the HCP atlas.

Statistic type for inference (See Eklund et al. 2016): ROI-level

Correction: FDR

Models & analysis

n/a	Involved in the study
☒	☐ Functional and/or effective connectivity
☒	☐ Graph analysis
☐	☒ Multivariate modeling or predictive analysis

Multivariate modeling and predictive analysis

For each ROI and each event, its activity pattern was quantified as a vector of multi-voxel normalized betas by dividing the original betas by the square root of the covariance matrix of the error terms from the GLM estimation. All voxels in the ROIs were used in the calculation of pattern similarity. Pattern similarity was measured as Fisher transformed Pearson's r. All pattern similarity analyses were conducted across runs, in order to avoid artifacts. Multiple comparisons were controlled for using FDR correction. Unless otherwise specified, all reported P-values were uncorrected.

Trial-level reinstatement was measured using the match/mismatch contrast on the right panel of Fig. 4B. The Different CTD condition was excluded from this analysis to avoid the measure of CTD reinstatement being confounded by the mismatch of CTD between the two contexts (e.g., the mismatch of CTD may impact the context-trial pattern similarity and/or its congruency effect). Trial-level reinstatement was then used to predict trial-level RT. Temporal model prediction error, task, and ROI-mean univariate activity were used as regressors of no interest.

We examined whether hippocampal context-context pattern similarity and univariate activity predicted frontoparietal cortical reinstatement of the CTD. Specifically, a block-wise analysis was conducted for each of the four cortical ROIs showing CTD reinstatement effects (Fig. 4C-F). For each ROI, hippocampal context-context pattern similarity in Same Context, Same CTD and Different CTD conditions, along with the hippocampal univariate activity were used as four predictors of block-wise CTD reinstatement in the ROI (calculated using the contrast on the right panel of Fig. 4B); the univariate activity of the frontoparietal ROI was used as a co-variate of no interest.
